# T cell activity in successful treatment of chronic urticaria with omalizumab

**DOI:** 10.1186/1476-7961-9-11

**Published:** 2011-07-26

**Authors:** Inmaculada Sánchez-Machín, Javier Iglesias-Souto, Andrés Franco, Yvelise Barrios, Ruperto Gonzalez, Víctor Matheu

**Affiliations:** 1 Alergología, Hospital del Tórax (Ofra); Complejo Hospitalario Universitario NS Candelaria, S/C Tenerife, Spain; 2 Immunology Section, Central Lab, Hospital Universitario de Canarias, La Laguna, Spain; 3 Department of Clinical Sciences-Division IV, Lund University, Lund, Sweden; 4 Research Unit; Complejo Hospitalario Universitario NS Candelaria, S/C Tenerife, Spain; 5 Research Unit, Hospital Universitario NS Candelaria, Ctra. Rosario 145, S/C Tenerife, 38010 Spain; 6 Department of Clinical Sciences, Division IV, Lund University, Lund 22185 Sweden

## Abstract

Omalizumab, a humanized monoclonal anti-IgE antibody has the potential to alter allergen processing. Recently, it has been postulated the assessment of PHA-stimulated adenosine triphosphate (ATP) activity as maker of CD4+ T cells activity in peripheral blood cells. We present the case report of a 35-year-old woman with a history of chronic idiopathic urticaria and angioedema of 8 years of development with poor response to treatment. The patient was partially controlled with cyclosporine at doses of 100 mg/12 h. However, she was still developing hives daily. Finally treatment with omalizumab was started at dose of 300 mg every 2 weeks. The patient experienced a decrease in urticarial lesions 2 days after starting therapy. We also evaluated the effects of omalizumab therapy on the activity of peripheral blood CD4+ T cells from the patient, in order to determine the potential modification of anti-IgE therapy on the process of antigen presentation-recognition. Activity of CD4+ cells by ATP release was clearly increased demonstrating an enlarged CD4 activity. Omalizumab may be useful in the treatment of severe chronic urticaria. ATP activity of peripheral blood CD4+ T cells might be a non-subjective method to assess Omalizumab activity.

## 

We have read the interesting manuscript recently published in Clinical and Molecular Allergy entitled "Down regulation of the high-affinity IgE receptor associated with successful treatment of chronic idiopathic urticaria with omalizumab" [1]. The study demonstrated the effectiveness of omalizumab in treating chronic idiopathic urticaria and the temporal relationship between improvement and down regulation of the high affinity IgE receptor (FcεRI). Omalizumab is a recombinant humanized monoclonal antibody that blocks free-serum immunoglobulin E (IgE) through the high-affinity Fc receptor from attaching to mast cells and prevents IgE-mediated inflammatory changes [2]. The FDA approved only specific indications for omalizumab use including patients older than 12 years with moderate-persistent to severe-persistent asthma with a positive skin test or in vitro reaction to a perennial aeroallergen and be symptomatic with inhaled corticosteroids.

However, anti-IgE appears to provide a therapeutic option for cases of many allergic diseases and conditions in which IgE plays a significant role. Although, the potential use of omalizumab in other IgE-mediated conditions is being investigated [3,4] and trials in allergic rhinitis are running, omalizumab is currently been evaluated for treating food allergy including peanut allergy, latex allergy, atopic dermatitis, and chronic urticaria [3,5,6].

We would like to present a 35-year-old woman with findings of rhinoconjunctivitis and episodic asthma by mite sensitization from childhood, severe chronic urticaria and angioedema since November 1999 with normal initial study conducted in 2000 (biochemistry, haemotology, serology and microbiology analysis). Poor control was obtained with conventional treatments (antihistamines and oral corticosteroids). Subsequently, the patient consulted several specialists (dermatologists) without success and was re-evaluated by Allergology during hospitalization caused by severe urticaria angioedema exacerbation coincident with an episode of retinal detachment. In previous years the urticaria and angioedema had not changed and she still had symptoms daily. Only in 2004 during pregnancy and subsequent breastfeeding showed a slight improvement in their symptoms.

A new study was done with normality of all the tests, including complement proteins study again. Then, we tried different treatments with antihistaminics, doxepin and corticosteroids. In April 2005, we began cyclosporine at doses of 200 mg per day with good response initially. Despite of oral contraception method the patient had a spontaneous miscarriage in that year. During the next 4 years the minimal doses of cyclosporine were of 100 mg per day and the last 2 years with daily cutaneus lesions. The pacient had exacerbations after walking, exposure to cold, premenstrual phase and the laboral absenteeism were important. Due to the poor control obtained previously, we decided to initiate Omalizumab therapy in 2008 with 300 mg every 2 weeks, based on weight and IgE level (178.0 UI/ml). Dramatic relief was obtained within 72 hours. The patient discontinued by own decision all medication with no exacerbation. Two weeks later, she had not injuries and did not take any medication. We began to gradually increase the intervals between doses. Currently we give 300 mg every 6 weeks and the patient remains asymptomatic without any side effects. Further, we tried to extend it to 8 weeks, but resulting with small hives in patient's extremities.

In parallel, whole blood was obtained before each administration for 18 weeks. Peripheral blood mononuclear cells (PBMC) were obtained and used in fresh for an immune cell function assay to detect T cell activation (ImmunoKnow™, Cylex Inc. Columbia, MD). Briefly, PBMC were incubated 18-h either in the absence of stimulant to assess basal activity or with specific stimulant for T cells (phytohemagglutinin-PHA). Magnetic beads coated with mouse monoclonal anti-human CD4 (Dynabeads^® ^CD4, Dynal Biotech A.S.A., Oslo, Norway) were added to immunoselect CD4 cells from both the stimulated and non-stimulated cells. After washing the selected CD4 cells on a magnet tray, a hypotonic basic solution as lysis reagent was added to release intracellular ATP. During incubation, increased ATP synthesis occurs within the cells that respond to PHA. The ATP result was measured by luminescence (562 nm). Serum obtained was stored and total IgE (UniCAP^®^, Phadia, Uppsala, Sweden) of every sample were determined in the same immunoassay with no significant differences among samples. However, T cell activation was significantly increased from basal (365 ng/ml ATP-moderate response) to first point, 2 weeks after first injection (593 ng/ml-strong response). That activation was maintained during following 18 weeks (Figure 1).

**Figure 1:**
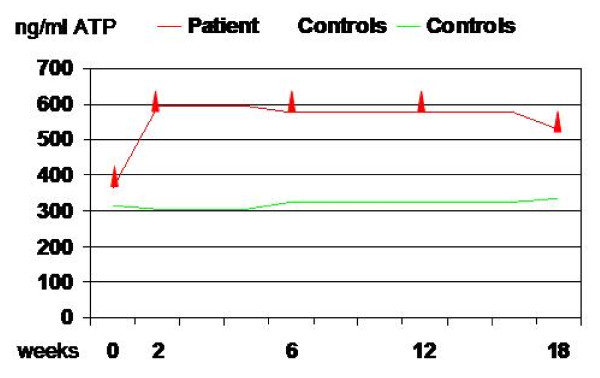
**Serial Follow up of T cell activity**. Serial Follow up of T cell activity measured as ATP activity release from T-cell during 18 weeks; patient (red line); control (green line).

The mechanism of action of omalizumab, an anti-IgE monoclonal antibody, in urticaria [7] is unknown, but in asthma act inducing the downregulation of IgE receptors [8,1]. Moreover, omalizumab produces a down regulation of IgE-mediated basophil activity [9,10] and a modification of the functional characteristics of dendritic cells [8]. CD4+T cells have a pivotal role in the process of antigen recognition in the adaptative inmune response. Recently, it has been postulated the assessment of PHA-stimulated adenosine triphosphate (ATP) activity as maker of CD4+T cells activity in peripheral blood cells [11]. We evaluated the effects of omalizumab therapy and observed the successful response to low doses of omalizumab in recalcitrant chronic urticaria and follow up using peripheral blood CD4+ showing an increase in activity by measurement of ATP release. ATP activity of peripheral blood CD4+T cells might be a non-subjective method to assess omalizumab activity [12], since the lack of other objective laboratory test. Further observations are needed.

## Competing interests

The authors declare that they have no competing interests.

## Authors' contributions

ISM & JIS studied the case report and wrote the initial draft of the manuscript. AF & YB performed every single lab assay. for in vivo tests. RG was responsible for the Drug Allergy Section and for safety of administration with Omalizumab. VM & YB conceived the idea and are responsible of the final version of the manuscript. All authors approved the final version of the manuscript.
